# Determinants of nursing students’ satisfaction with blended learning

**DOI:** 10.1186/s12912-024-02393-y

**Published:** 2024-10-18

**Authors:** Eman Arafa Hassan, Ahlam Mahmoud Mohamed, Fatma Abdou Eltaib, Asmaa Mohammed Saad Khaled

**Affiliations:** 1 Critical Care and Emergency Nursing Department, Faculty of Nursing, Alexandria University, Alexandria, Egypt; 2 Community Health Nursing, Faculty of Nursing, Alexandria University, Alexandria, Egypt; 3 Faculty of Nursing, Ain Shams University, Cairo, Egypt

**Keywords:** Blended learning, Determinants, Nursing students, Satisfaction

## Abstract

**Background:**

Blended learning, a pedagogical approach combining traditional classroom instruction with online components, has gained prominence in nursing education. While offering numerous benefits, student satisfaction with blended learning remains a critical concern. This study contributes to the existing literature by providing a comprehensive evaluation of the determinants influencing nursing students’ satisfaction with this innovative educational modality. By examining a wide range of factors, including sociodemographic characteristics, academic factors, and environmental influences, this research offers valuable insights for educators to optimize blended learning experiences in nursing education.

**Methods:**

A descriptive cross-sectional research design was conducted. This study investigates the factors influencing nursing students’ satisfaction with blended learning at Alexandria University, Egypt, where blended learning programs have been integrated into the curriculum primarily through the Microsoft Teams platform. A convenient sample of 1266 nursing students from both bachelor and technical educational institutions participated in the study from September 2023 to the end of December 2023. Data were collected using an online survey containing two measurement tools: the Blended Learning Satisfaction Scale and the Environmental Facilitators and Barriers to Student Persistence in Online Courses scale. Statistical analyses, including descriptive statistics and backward multiple linear regression, were conducted to identify factors that are associated with the satisfaction of nursing students’ with blended learning.

**Results:**

Findings indicate that factors such as age, gender, income, employment status, access to suitable internet sources, academic year, computer literacy, preferred learning method, and perceptions of environmental facilitators significantly influence satisfaction scores (all *p* < 0.001). The overall regression model, with an adjusted R² of 0.31, signifies that 31% of the variance in satisfaction scores is explained collectively by the previously mentioned variables (F = 21.21, *p* < 0.001).

**Conclusion:**

Students’ sociodemographic variables, preference for blended learning, and perception of environmental facilitators such as encouragement to enroll in the course significantly influence nursing students’ satisfaction levels with blended learning. However, limitations in the current study such as self-report bias, convenient sampling, and cross-sectional design limit the generalizability and causal inferences of these findings.

**Supplementary Information:**

The online version contains supplementary material available at 10.1186/s12912-024-02393-y.

## Introduction

In the rapidly evolving landscape of higher education, universities globally are embracing virtual learning environments, leading to the widespread adoption of blended learning that combines online and face-to-face instruction [1]. The integration of learning management systems and the use of information and communication technologies have become integral aspects of students’ lives [2, 3]. The transformative impact of technology on education was further accelerated by the global pandemic, compelling institutions to swiftly shift to distance and online learning due to the imposition of national lockdowns [4, 5].

Blended learning manifests in various forms, including web courses, web enhancement courses, and web-centric courses [6]. Each method has its unique characteristics, blending online and face-to-face elements to different extents [7]. The role of educators in the blended learning model assumes a multifaceted dimension, encompassing roles as facilitators, motivators, mentors, and counselors. This approach emphasizes a collaborative learning environment where teachers act as friends, both online and offline, fostering an open and flexible learning experience aligned with students’ needs [8, 9].

In the context of nursing education, blended learning integrates theoretical knowledge with practical application, utilizing a variety of resources such as virtual simulations, interactive modules, and instructor-led discussions [10]. This approach not only accommodates diverse learning styles but also fosters self-directed learning and critical thinking skills essential for nursing practice in today’s complex healthcare environment [11]. By seamlessly blending technology with traditional classroom instruction, blended learning in nursing education enhances accessibility, flexibility, and engagement, ultimately contributing to students’ satisfaction and competency development [12].

The significance of understanding the determinants of nursing students’ satisfaction with blended learning becomes paramount. Blended learning necessitates the effective utilization of technology, considers learner characteristics, and relies on participants’ commitment [13, 14]. Factors such as computer competency, social and family support, workload management, age, gender, and attitude emerge as crucial elements in the context of higher educational institutions [15]. Moreover, the innovative pedagogy and instructional design supporting blended learning emphasize its potential to reshape traditional education paradigms [16, 17].

Previous studies have acknowledged the multifaceted nature of student satisfaction, emphasizing the intricate interplay of diverse factors. The literature suggests that factors such as instructional design, course content relevance, and the quality of online and face-to-face interactions significantly influence student satisfaction in blended learning environments [15, 18]. Additionally, the perceived effectiveness of assessment methods and the alignment of learning objectives with students’ professional goals have been identified as critical components shaping students’ satisfaction in blended learning settings [15, 19].

In this dynamic educational experience, student satisfaction has emerged as a critical concern for higher education sponsors operating in an increasingly competitive market. It has become an integral component of quality assurance and quality enhancement efforts [20, 21]. The level of learner satisfaction, reflecting attitudes and feelings towards the advantages of blended learning classrooms, plays a pivotal role in gauging the effectiveness of this educational approach [22].

Despite the wealth of research exploring general student satisfaction in blended learning, there remains a discernible gap in the specific context of nursing education. Nursing students constitute a unique cohort with distinct educational needs and professional expectations. The scant literature addressing nursing students’ satisfaction with blended learning emphasizes the need for a targeted investigation into the determinants that resonate within this specialized field. Understanding the factors that influence nursing students’ satisfaction with blended learning is crucial for educational institutions striving to tailor their programs to meet the evolving demands of the healthcare sector [23, 24]. Therefore, this study aims to meticulously assess the determinants of nursing students’ satisfaction with blended learning, contributing valuable insights to the ongoing discourse on the effectiveness and optimization of blended learning methodologies in nursing education.

## Method

### Aim

This study aimed to comprehensively evaluate the determinants influencing nursing students’ satisfaction with blended learning.

### Design

A cross-sectional research design was applied in this study.

### Settings

This study was conducted within the academic institutions of Alexandria University, specifically the Faculty of Nursing and the affiliated Technical Institute of Nursing in Alexandria, Egypt. The Faculty of Nursing at Alexandria University stands as a center for nursing education, fostering academic excellence and professional development. The Technical Institute of Nursing, closely affiliated with the university, complements this academic ecosystem by providing specialized technical training in nursing.

Within this dynamic academic setting, blended learning has been seamlessly integrated into the educational fabric, predominantly utilizing the Microsoft Teams platform. Blended learning methods, such as web courses, web enhancement courses, and web-centric courses, have become prevalent, enhancing the learning experience for nursing students. Microsoft Teams serves as a versatile platform for online lectures, offline lectures, assignments, quizzes, and video resources supporting nursing education. This integration highlights the commitment to providing a comprehensive and interactive learning environment that seamlessly combines face-to-face and online elements.

### Participants

A convenient sample of 1266 nursing students from both bachelor and technical educational institutions were included in this study, representing diverse cohorts across different semesters. The inclusion of students from various semesters ensures a comprehensive understanding of satisfaction determinants throughout the academic progression. The exclusion criteria were students who are not currently enrolled in nursing programs or those who do not consent to participate in the study. All nursing students included in this study, both bachelor’s and technical nursing education students, had completed at least one blended nursing course. By September 2023, the start of data collection for this study, all nursing students affiliated with Alexandria University had completed at least one nursing course with a blended learning component. Blended learning was initially adopted in response to the COVID-19 pandemic and has continued to be integrated into many nursing courses at Alexandria University.

Power Analysis and Sample Size (PASS) program version 20 was employed for sample size estimation, incorporating a power analysis. The minimum sample size was determined based on a power of 90%, a level of significance set at 0.05, and a minimum sample size of 1000. To enhance precision, a moderate effect size, based on prior research [25, 26] and expert judgment, was included in the calculation. Additionally, expected variability, crucial for accurate sample size estimation, was considered. The chosen multivariate regression model, accounting for multiple predictors, was also factored into the estimation. Potential dropout or non-response rates were carefully considered to ensure the study’s robustness against these challenges.

### Measurement tools

In the current study, we utilized two standardized tools, with permission, in their original English versions. Tool one was used to assess nursing students’ satisfaction with blended learning [27], while tool two aimed to evaluate the environmental facilitators and barriers affecting persistence in blended learning courses, particularly in its online aspect [28]. These tools were previously validated in their original studies. Furthermore, they were revalidated in the current study to ensure their relevance to the study’s aims and appropriateness for Egyptian nursing students. This revalidation followed a pilot study involving 56 Egyptian nursing students and incorporated evaluations from eleven nursing education experts regarding the tools.

Tool one is “Blended Learning Satisfaction Scale (BLSS)”. This scale was developed by Zeqiri et al. (2021) [27] to assess nursing students’ satisfaction with blended learning. This scale comprises thirteen statements across four domains: course management (four statements), interaction (three statements), performance (three statements), and satisfaction (three statements). Statements are rated on a 5-point Likert scale, one (strongly disagree) to five (strongly agree). The total score ranges from 13 to 65, with a higher total score representing a higher level of satisfaction.

The scale internal consistency of Cronbach’s test ranges from 0.715 to 0.931 in its original report [27]. We assessed whether the tool effectively measures the theoretical constructs it is intended to evaluate within the Egyptian context. Utilizing confirmatory factor analysis (CFA), we found that all items had factor loadings exceeding 0.5, demonstrating a strong positive correlation with their underlying constructs. Additionally, we ensured face and content validity, achieving a content validity index greater than 0.90 for each domain. The reliability of the tool, as measured by Cronbach’s alpha, ranged from 0.78 to 0.92 in this study.

Tool two is “The environmental Facilitators and Barriers to Student Persistence in Online Courses”. This tool was developed by Heilporn and Lakhal (2022) [28] to measure the environmental facilitators and barriers encountered by students. This tool, originally designed for assessing student persistence in online courses, was adapted to the context of blended learning for this study.

The tool is composed of 16 items and utilizes a 5-point Likert scale used to rate each statement coded from 1 “strongly disagree” to 5 “strongly agree”. The total score ranges from 16 to 80, with a higher total score representing a highly perceived facilitator to continue in the courses. It consists of four domains: Encouragements (five statements), Time to Events (four statements), Potential Dropout (five statements), and Cost-Benefit (two statements). While the tool’s original focus was on online courses, many of its statements, such as those related to encouragements, time-events, and potential dropout, are directly relevant to the facilitators and barriers that nursing students might face in a blended learning environment.

In a prior study, the tool’s Cronbach’s alpha ranged from 0.79 to 0.87 ^28^. In the current study, we conducted a CFA to ensure that this tool effectively captures the constructs of blended learning facilitators and barriers within the Egyptian blended learning context. The analysis revealed factor loadings exceeding 0.62 for each item, which is considered acceptable. Additionally, we performed a principal component analysis (PCA) to verify the underlying structure of the domains. The PCA confirmed the suitability of the four domains of facilitators and barriers, with eigenvalues exceeding one and explaining a cumulative variance of 58.27% of the total variance. Moreover, the content validity index of the tool was 0.92, and the Cronbach’s alpha reliability for all domains ranged from 0.82 to 0.89.

The socio-demographic characteristics of students (e.g., age, gender, income, residence working, level of study, academic year, CGPA), as well as the availability of suitable internet sources and suitable electronic device on which the students studied, computer literacy, and preferable method of learning, were attached to the survey. The survey is provided as a supplementary file S1.

### Data collection

Data collection was started from the beginning of September 2023 to the end of December 2023. The researchers communicated with the selected students over the phone and a voice message was sent on WhatsApp groups to explain the aim of the study. The data was collected through sharing a questionnaire using online Google Forms, and the questionnaire link sent among specific WhatsApp application groups for communication between students. The researchers asked the team leaders to help in sharing the questionnaire link among their WhatsApp groups. To reduce the missing data, the students were mandatory to fill all the items in the online questionnaire or else could not reach the next page; a notification box indicating a warning reminder that one or more items were not answered. After completing the questionnaire, the students were directed to click the submitted option and finally, the online questionnaire was sent to the drive.

### Ethical considerations

Approval from the Research Ethics Committee of the Faculty of Nursing, Alexandria University, was obtained (Institutional Review Board: IRB00013620). After explaining the study’s purpose, all participants were provided with informed written online consent for participation by clicking agree to participate button at the beginning of the electronic questionnaire. The researchers emphasized participants’ rights to voluntarily participate, refuse, or withdraw from the study at the beginning of the online questionnaire. To ensure data confidentiality, the online survey was conducted anonymously. No personal identifiers such as names, codes, or email addresses were required in the online forms. The collected data were stored on a secure, password-protected Google drive that is not accessible to unauthorized individuals. Only authorized research team members had access to the collected data. This work has been carried out in accordance with The Code of Ethics of the World Medical Association (Declaration of Helsinki) on Human participants.

### Statistical analysis

The Statistical Package for Social Sciences (SPSS version 28) was utilized for both data presentation and statistical analysis. Descriptive statistics, including number, percentage, means, and standard deviation (SD), were used to describe the socio-demographic characteristics, students’ satisfaction with blended learning, and environmental facilitators and barriers to student persistence in online courses. A backward multiple linear regression model was used to identify factors associated with nursing students’ satisfaction with blended learning. All the statistical analyses were considered significant at *P* *≤* 0.05.

## Results

Table 1 illustrates the sociodemographic and learning characteristics of the 1266 nursing student participants, offering insights into the composition of the study’s sample. The mean age of the students was 20.47 years, demonstrating a relatively homogeneous age distribution with a low standard deviation (± 1.82). The gender distribution reveals a notable majority of female students (67.5%) compared to their male counterparts (32.5%). In terms of residency, approximately two-thirds of the participants reside in urban areas (64.8%), while the remaining third is from rural backgrounds (35.2%). Financially, 58.1% of students report having enough income, indicating a measure of financial stability within the cohort. A significant portion of the students (59.7%) is engaged in employment, suggesting a balance between academic pursuits and work responsibilities.

**Table 1:** Nursing students’ sociodemographic and learning variables (*n* = 1266)

Students’ variables	*N* (%) or Mean ± SD
Age	20.47 ± 1.82
**Gender**	
Male Female	411 (32.5%) 855 (67.5%)
**Residency**	
Urban Rural	820 (64.8%) 446 (35.2%)
**Income**	
Enough Not enough	735 (58.1%) 531 (41.9%)
**Working**	
Yes No	756 (59.7%) 510 (40.3%)
**Availability of suitable internet source**	
Yes No	1218 (96.2%) 48 (3.8%)
**Availability of suitable electronic device to study on**	
Yes No	1183 (93.4%) 83 (6.6%)
**Level of study**	
Technical degree Bachelor’s degree	414 (32.7%) 852 (67.3%)
**Cumulative Grade Point Average (CGPA)**	3.64 ± 2.71

Examining the learning environment and resources, it is noteworthy that an overwhelming majority of students (96.2%) have access to a suitable internet source, emphasizing the ubiquity of internet connectivity among the participants. Additionally, a substantial percentage (93.4%) possess suitable electronic devices for study purposes, ensuring accessibility to online learning resources. In terms of academic pursuits, the majority of students are pursuing a Bachelor’s degree (67.3%), while the remaining 32.7% are enrolled in technical degree programs. The mean Cumulative Grade Point Average (CGPA) of 3.64 ± 2.71 provides an overview of the academic performance of the participants, reflecting a moderate level of achievement.

**Fig. 1:**
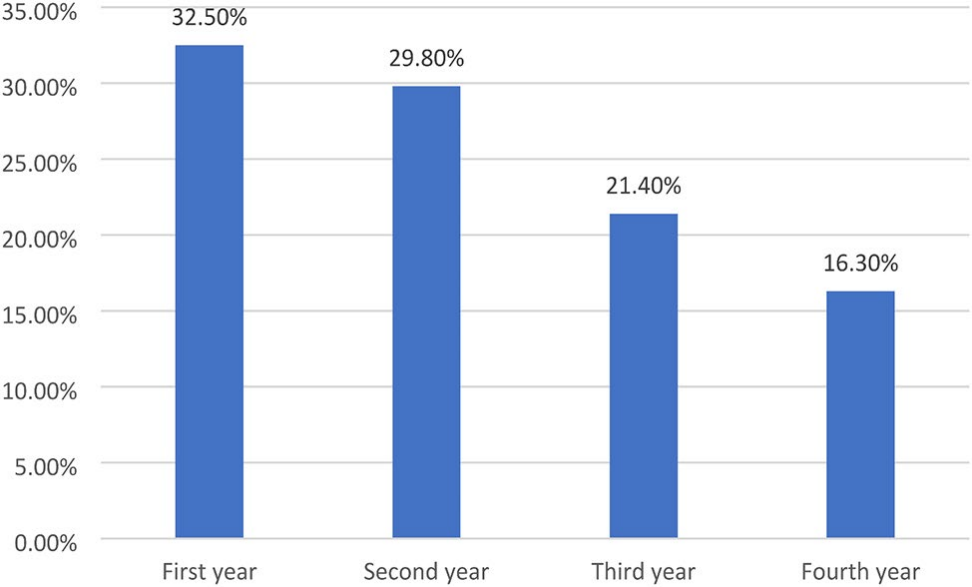
Distribution of students according to their academic year

**Fig. 2:**
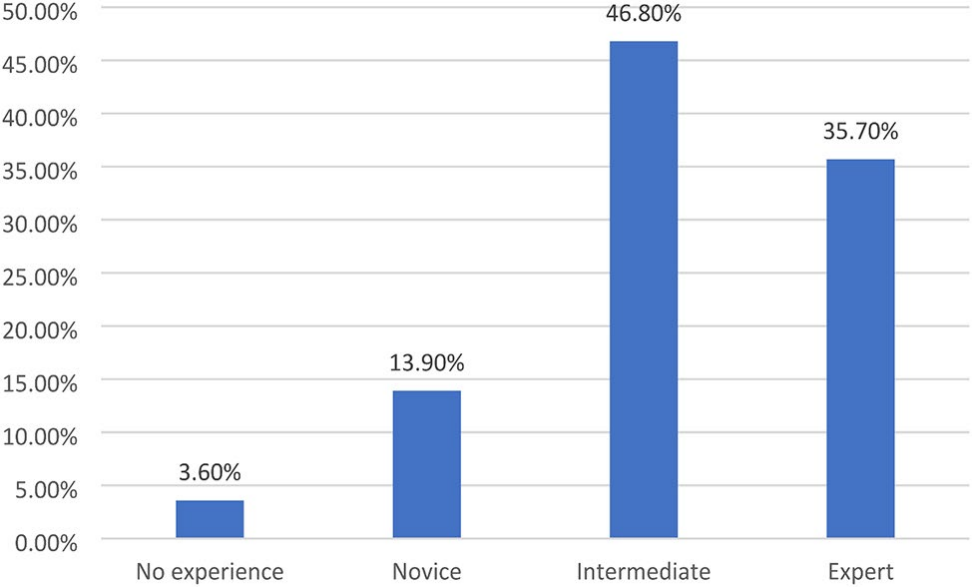
Distribution of students according to their computer literacy

**Fig. 3:**
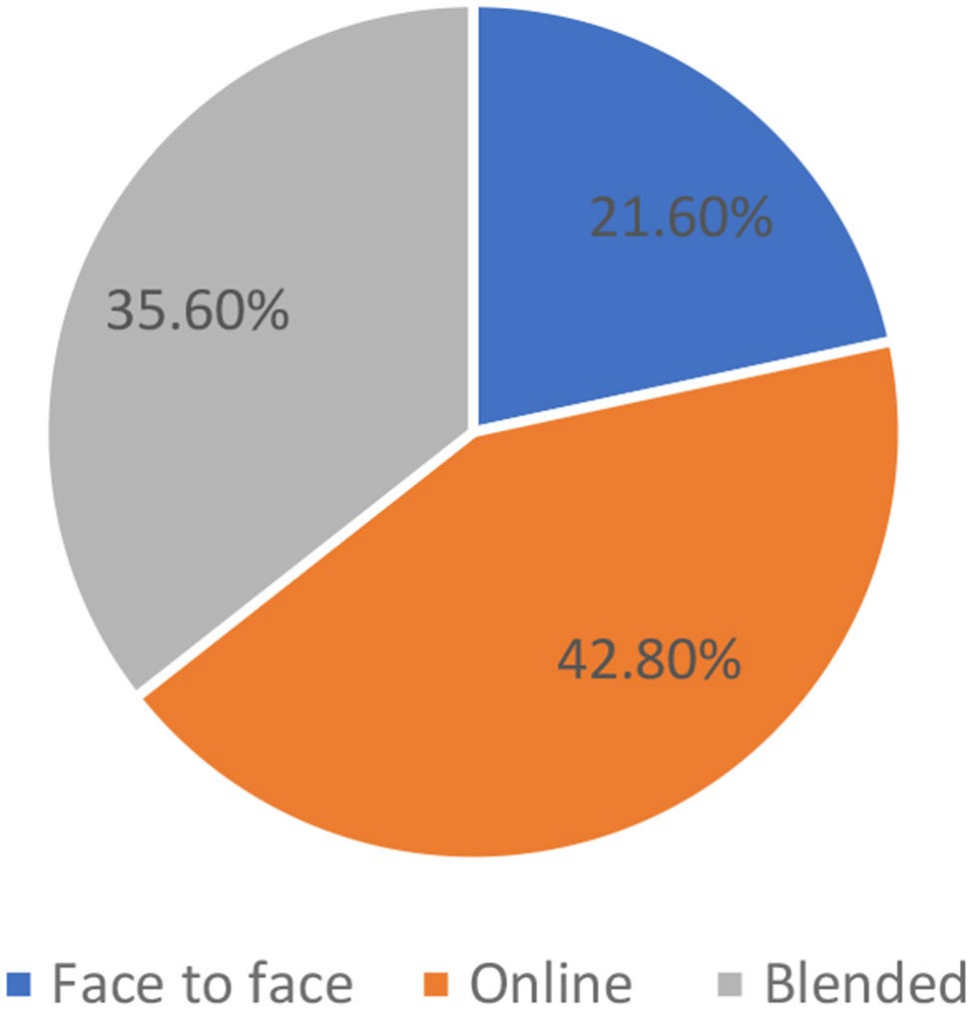
Distribution of students according to their preferable method of learning

The distribution of students in Fig. 1 across academic years demonstrates a balanced representation, with the highest proportion in the first year (32.5%) and a gradual decline in subsequent years. Concerning technology proficiency, Fig. 2 shows that the majority of students report intermediate computer literacy (46.8%), while a notable percentage claim expertise (35.7). Lastly, Fig. 3 revealed that the preferred method of learning among participants is diverse, with a significant interest in online learning (42.8%) and blended learning (35.6%), alongside a smaller preference for face-to-face instruction (21.6%).

Table 2 provides an overview of nursing students’ satisfaction with various aspects of blended learning, with mean scores and standard deviations indicating the level of agreement or disagreement. Examining specific satisfaction items, the data reveals that student’s rate of “Interaction during the courses” is the highest, with a mean score of 3.62 ± 0.88. This relatively low standard deviation suggests a consensus among students regarding positive interaction experiences. On the other hand, “Course Management” and “Performance in the courses” receive lower mean scores of 2.69 ± 1.01 and 2.78 ± 1.08, respectively, accompanied by higher standard deviations, indicating more varied opinions. The overall “Satisfaction with blended learning” mean score of 3.21 ± 0.93 suggests a moderate level of contentment, while “Overall satisfaction” with a mean score of 3.07 ± 0.49 and a low standard deviation indicates a relatively consistent level of satisfaction across students.

**Table 2:** Students rating of blended learning satisfaction (*n* = 1266)

Satisfaction items	Mean score	Standard deviation
Course Management	2.69	1.01
Interaction during the courses	3.62	0.88
Performance in the courses	2.78	1.08
Satisfaction with blended learning	3.21	0.93
**Overall satisfaction**	3.07	0.49

Table 3 shows nursing students’ perceptions of environmental facilitators to blended learning, offering insights into factors that contribute to their learning experience. Notably, students express a high level of agreement with “Encouragements to enroll in blended learning courses,” as evidenced by a mean score of 3.99 and a moderate standard deviation of 0.85, highlighting positive support structures. Additionally, the flexibility of “Time and events of blended learning courses” is acknowledged, though with a lower mean score of 3.31 and a higher standard deviation of 0.98, indicating some variability in opinions. Students perceive the “Potential of dropout in blended learning” and the “Cost-benefit of blended learning courses” with mean scores of 3.48 ± 1.08 and 3.70 ± 0.01, respectively, suggesting moderate agreement with these factors. The overall agreement on facilitators and barriers, with a mean score of 3.62 and a low standard deviation of 0.47, indicates a generally consistent viewpoint among students regarding the factors influencing their blended learning experience.

**Table 3:** Students rating of environmental facilitators to blended learning (*n* = 1266)

Environmental facilitator	Mean	Standard deviation
Encouragement to enroll in the courses	3.99	0.85
The time and events of blended learning courses are flexible	3.31	0.98
The potential of dropout in blended learning	3.48	1.08
Cost-benefit of blended learning courses	3.70	0.01
**Overall agreement on facilitators and barriers**	3.62	0.47

**Fig. 4:**
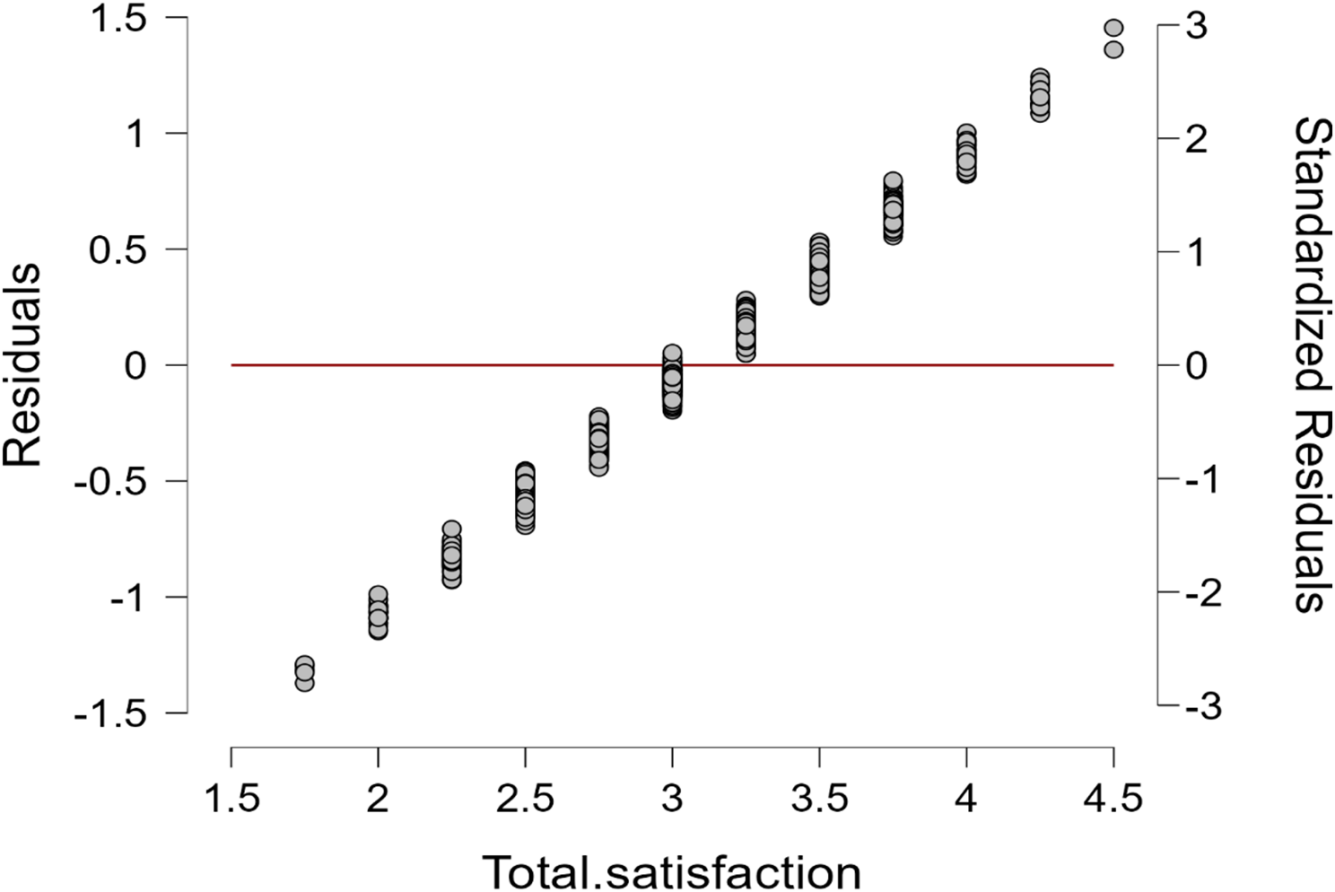
Scattered plot of multiple linear regression for estimated against the students’ blended learning satisfaction

Figure 4 of the scattered plot of multiple linear regression provide the predictor estimated against the students’ blended learning satisfaction. The plots in linear line indicating all the predictors involved in this model have p-values less than 0.05, indicating they are statistically significant in predicting satisfaction scores.

The multiple linear regression model of Table 4 identified factors influencing nursing students’ satisfaction with blended learning. Age exhibits a modest negative association with satisfaction scores (B = -0.04, *p* = 0.048), suggesting that, within the 95% confidence interval (-0.06 to -0.03), as students’ age increases, satisfaction marginally decreases. Female students express higher satisfaction (B = 0.25, *p* = 0.037) compared to their male counterparts, with a 95% confidence interval of 0.34 to 0.86. Income has a significant negative impact (B = -0.88, *p* = 0.033), indicating that, within the 95% confidence interval (-0.98 to -0.26), lower income is associated with lower satisfaction scores.

**Table 4:** Summary of multiple linear regression model for satisfaction score using the standard model

Variables	B	SE	*P*	95% CI
Age	-0.04	0.208	0.048	-0.06 to -0.03
Gender	0.25	0.131	0.037	0.34 to 0.86
Income	-0.88	0.229	0.033	-0.98 to -0.26
Working	0.62	0.190	0.040	0.11 to 0.74
Availability of suitable internet source	0.45	0.291	0.029	0.53 to 0.80
Academic year	0.47	0.210	0.028	0.18 to 0.61
Computer literacy	0.90	0.385	0.015	0.89 to 1.41
Preferable method of learning	1.11	0.445	0.006	1.02 to1. 44
Encouragement to join blended learning courses	0.46	0.320	0.010	0.05 to 0.65
Time and events of blended learning	0.52	0.119	0.035	0.21 to 0. 54
Cost-benefit of blended learning	0.42	0.218	0.013	0.08 to 0.60

Adjusted R^2^ = 0.31

F = 21.21

*P* < 0.001

Conversely, employed students (B = 0.62, *p* = 0.040) exhibit higher satisfaction, possibly due to a sense of accomplishment or additional resources gained from employment. Access to a suitable internet source (B = 0.45, *p* = 0.029) positively influences satisfaction, with a 95% confidence interval of 0.53 to 0.80, emphasizing the importance of internet accessibility. Higher academic years (B = 0.47, *p* = 0.028), within a 95% confidence interval of 0.18 to 0.61, and computer literacy (Std beta = 0.90, *p* = 0.015), within a 95% confidence interval of 0.89 to 1.41, are both linked to increased satisfaction scores, highlighting positive adaptation and technological proficiency.

Notably, students who prefer blended learning (B = 1.11, *p* = 0.006), within a 95% confidence interval of 1.02 to 1.44, and receive encouragement to enroll (B = 0.46, *p* = 0.010), within a 95% confidence interval of 0.05 to 0.65, demonstrate significantly higher satisfaction. The flexibility of time and events in blended learning (B = 0.52, *p* = 0.035), within a 95% confidence interval of 0.21 to 0.54, positively influences satisfaction, as does the perceived cost-benefit of blended courses (B = 0.42, *p* = 0.013), within a 95% confidence interval of 0.08 to 0.60.

The overall model, with an adjusted R² of 0.31, signifies that 31% of the variance in satisfaction scores is explained collectively by the included variables. The statistically significant F-value of 21.21 (*p* < 0.001) highlights the overall significance of the model, reinforcing its predictive power.

## Discussion

Blended learning has become a dynamic educational approach, combining traditional face-to-face instruction with online elements, creating a flexible and interactive environment for students [12]. This study assessed nursing students’ satisfaction with blended learning in academic institutions. The assessment covers various aspects, from students’ perceptions of course interactions and management to their understanding of environmental facilitators. Additionally, we use a robust multiple linear regression model to analyze the complex network of factors affecting satisfaction. The ultimate goal is to offer practical insights for educators, institutions, and policymakers looking to enhance blended learning experiences for nursing students, promoting a more personalized and responsive approach to modern pedagogy.

In assessing nursing students’ satisfaction with blended learning, the findings presented in the current study offer insights into various dimensions of their blended satisfaction experience. Notably, students uniformly express a positive attitude toward interaction during the courses, suggesting a consensus on the significance of engaging interactions. This aligns with existing literature highlighting the crucial role of interaction in fostering a sense of collaborative learning in online and blended environments [29, 30].

Conversely, course management and performance in the courses elicit lower satisfaction scores, coupled with higher variability among responses. This suggests diverse opinions and areas for potential enhancement. The reasons behind this variation may be explained by the fact that students came from different nursing programs, such as bachelor’s and technical nursing programs, each with distinct experiences. These differences likely result in varying levels of satisfaction with course management and performance in blended learning environments. This aligns with findings from studies comparing student satisfaction across different programs, which emphasize how program structure impacts the blended learning experience [31, 32].

Moreover, the literature acknowledges the challenges associated with effective course management and maintaining performance standards in online settings [33, 34]. These studies point to the need for targeted improvements in these domains to enhance overall satisfaction. The moderate overall satisfaction level in the current study reflects a balanced sentiment among nursing students. This finding is consistent with research suggesting that well-implemented blended learning can provide a satisfactory educational experience [29, 35].

Concerning nursing students’ perceptions of environmental facilitators to blended learning, the current study presents a substantial agreement among students concerning encouragements to enroll in blended learning courses highlights the positive impact of institutional support structures. This aligns with a body of studies emphasizing the important role of encouragement and institutional backing in fostering student engagement in online and blended courses [36, 37]. The robust support for enrollment suggests that proactive measures to promote blended learning within academic institutions can significantly contribute to students’ favorable perceptions and satisfaction [36, 37].

Moreover, the acknowledgment of the flexibility in time and events of blended learning courses, although with some variability in opinions, echoes findings in existing literature recognizing the diverse needs of students regarding time management in online and blended learning environments [38, 39]. This highlights the importance of designing flexible course structures that accommodate varying schedules and preferences, catering to the individualized nature of student experiences [39]. While students express moderate agreement with concerns such as the potential of dropout in blended learning and the cost-benefit of blended learning courses, the overall low variability in responses suggests a generally consistent viewpoint among students in the current study. This aligns with studies investigating barriers to online education, emphasizing the need for institutions to address concerns related to dropout potential and cost-effectiveness to enhance the overall learning experience [40, 41].

The multiple linear regression model in the current study clarifies the intricate web of factors influencing nursing students’ satisfaction with blended learning. Particularly, the modest negative association between age and satisfaction scores suggests that as students’ age increases, satisfaction marginally decreases. This finding resonates with some existing literature highlighting potential challenges older students may face in adapting to technology-mediated learning [42]. Tailored support and interventions may be beneficial to enhance the satisfaction of older nursing students in blended learning environments [43].

On a gender-related note, the higher satisfaction expressed by female students aligns with a study suggesting that female students tend to engage more actively in online discussions and collaborative activities [44]. Understanding these gender dynamics could inform instructional strategies that cater to diverse learning preferences and participation levels [45]. Additionally, the significant negative impact of lower income on satisfaction highlights the socioeconomic factors influencing students’ experiences in blended learning. This is consistent with broader literature indicating that financial constraints can hinder access to resources and technology necessary for online education [46, 47]. Mitigating these disparities through targeted support mechanisms could contribute to a more equitable educational experience [48].

In the current study, the positive association between employment and satisfaction may be attributed to the flexibility of time between work and study that is offered by blended learning and limited in face-to-face learning [49]. This finding aligns with studies emphasizing the potential benefits of balancing work and study in enhancing satisfaction in online learning [13, 49]. Access to a suitable internet source emerges as an important determinant, positively influencing satisfaction. This aligns with a wealth of studies stressing the importance of digital infrastructure in online and blended learning environments [50, 51].

The positive links between higher academic years, computer literacy, and increased satisfaction scores highlight the role of positive adaptation and technological proficiency in fostering contentment. This supports existing studies emphasizing the importance of digital literacy in online and blended learning [52, 53]. However, it is important to note that the factors associated with blended learning satisfaction, such as computer literacy and access to technology, are only part of the equation. There may be additional unexplored variables, such as institutional infrastructure, technical support availability, and faculty preparedness, that contribute to student satisfaction. Addressing these factors in future research will be critical to fully understanding the drivers of satisfaction [13, 30].

### Future research directions

To address the variability in satisfaction scores, further studies should explore additional variables such as faculty development, technical support systems, and infrastructure investments. Comparative studies across different educational settings and longitudinal designs could provide a more nuanced understanding of how satisfaction evolves over time, and what specific interventions may improve satisfaction in different contexts.

### Implications of findings

The findings of this study emphasize the importance of considering the sociodemographic and learning characteristics of nursing students, in addition to the environmental facilitators and barriers, in fostering satisfaction with blended learning. Institutions aiming to enhance the experience of nursing students in blended learning environments should invest in robust faculty training programs, expand digital infrastructure, and provide socioeconomic support to students in need. Implementing these measures could significantly improve the personalized and responsive learning environment essential for modern nursing education.

### Strengths and limitations

Strengths of this study include its sample size of 1266 nursing students from diverse educational institutions ensures robustness and representativeness across different academic levels and cohorts. Rigorous statistical methods, including a power analysis and multivariate regression model, enhance the validity of the findings. The employment of established measurement tools, such as the Blended Learning Satisfaction Scale and the Environmental Facilitators and Barriers to Student Persistence in Online Courses scale, contributes to the study’s reliability.

This study has several limitations that should be acknowledged. The use of self-reported data may introduce response bias, as participants might provide inaccurate or biased answers. To mitigate this, surveys were anonymized to encourage honest reporting; however, response bias remains a potential concern. Additionally, the study employed a convenience sampling method, focusing solely on nursing students from Alexandria University, which may limit the generalizability of the findings to other populations of nursing students. The cross-sectional design of the study also prevents the establishment of causality between the determinants and satisfaction with blended learning, indicating a need for longitudinal studies to investigate the temporal relationships between these variables.

## Conclusion

Factors such as age, gender, income, employment status, access to suitable internet sources, academic year, computer literacy, preference for blended learning, and encouragement to enroll significantly influence nursing students’ satisfaction levels with blended learning. Notably, perceptions of flexibility in time and events, as well as the perceived cost-benefit of blended courses, play crucial roles in shaping satisfaction. These findings emphasize the importance of addressing diverse student needs and enhancing support structures to optimize satisfaction and effectiveness in blended learning environments.

## Electronic supplementary material

Below is the link to the electronic supplementary material.

Supplementary Material 1

## Data Availability

No datasets were generated or analysed during the current study.
