# Supplementary material for: Determinants of nursing students’ satisfaction with blended learning
Source: BMC Nurs. 2024 Oct 18;23:766. doi: 10.1186/s12912-024-02393-y (PMC11488250; doi:10.1186/s12912-024-02393-y)
Supplement: Supplementary file 1 — Supplementary Material 1 [file 12912_2024_2393_MOESM1_ESM.docx]

**Determinants of Nursing Students' Satisfaction with Blended Learning**

**Part 1: Students' Variables**

- Demographic Information:
  - Age (numerical)
  - Gender (categorical: Male, Female)
  - Residency (categorical: Urban, Rural)
  - Income (categorical: Enough, Not enough)
  - Working (categorical: Yes, No)
- Education Background:
  - Level of study (categorical: Technical degree, Bachelor degree)
  - Academic year (categorical: First year, Second year, Third year, Fourth year)
  - Cumulative Grade Point Average (CGPA) (numerical)
- Technology and Learning Preferences:
  - Availability of suitable internet source (categorical: Yes, No)
  - Availability of suitable electronic device to study on (categorical: Yes, No)
  - Computer literacy (categorical: No experience, Novice, Intermediate, Expert)
  - Preferable method of learning (categorical: Face-to-face, Online, Blended)

**Part 2: Blended Learning Satisfaction** (5-point Likert scale from strongly disagree to strongly agree)

- Course Management:
  - The online and face-to-face course components enhance and complement each other.
  - Online learning platforms are favorable for managing and organizing learning.
  - Blended learning makes it more convenient for arranging and grading assignments.
  - Blended learning makes it more convenient for publishing materials and information.
- Interaction:
  - Blended learning creates a user-friendly learning environment with teachers.
  - Blended learning improves the communication and interaction between students and teachers.
  - The use of blended learning technology encourages me to learn independently.
- Performance:
  - Blended learning improves my overall performance in courses.
  - I think blended learning is the best way of improving students’ performance.
  - I have better grades in classes that combine online and face-to-face instructions.
- Satisfaction:
  - I feel more satisfied when I study using blended learning.
  - I am more satisfied with this learning experience compared to traditional course settings.
  - I prefer a combined class with face-to-face and online instructions.

**Part 3: Environmental Facilitators and Barriers to Blended Learning** (5-point Likert scale from strongly disagree to strongly agree)

- Encouragements:
  - My partner encouraged me to enroll in this course.
  - My family encouraged me to enroll in this course.
  - My teachers encouraged me to enroll in this course.
  - My friends encouraged me to enroll in this course.
  - My spouse offered me his support so that I could study.
- Time-Events:
  - A change in my job has meant that I no longer have enough time to study.
  - A change in my work situation made it difficult to follow this course.
  - I was sick during this course, so it was difficult for me to keep a good pace.
  - Personal/family circumstances, which were not present at the time of registration, hindered my studies.
- Potential Dropout:
  - I am very determined to finish this course.
  - I often think about giving up this course.
  - I am undecided whether to finish this course.
  - I am about to drop this course.
  - I have already dropped this course.
- Cost-Benefit:
  - As I progress through this course, I continually weigh the pros and cons of the costs of staying in the program.
  - As I continue to enroll in courses, I always wonder if the cost financial "worth it" to continue.
